# Tuberculosis of the Male Genital Tract

**Published:** 1909-01-09

**Authors:** Lawrie McGavin

**Affiliations:** Surgeon to the Seamen's Hospital; Lecturer on Clinical Surgery in the London School of Clinical Medicine


### Hospital Clinics.

### TUBERCULOSIS OF THE MALE GENITAL TRACT.

By LAWRIE McGAVIN, F.R.C.S. Eng., Surgeon to the Seamen's Hospital; Lecturer on Clinical Surgery in the London School of Clinical Medicine.

(A Post-graduate Lecture delivered at the London School of Clinical Medicine.)

The subject of genital tuberculosis presents sufficient difficulty, not only of diagnosis and treatment, but perhaps especially of prognosis, to suggest it as one worthy of your consideration. The disease is comparatively common in all classes of life, and has as its chief characteristics insidiousness, a tendency to spread throughout the whole genital tract, great resistance to treatment, and a proneness to affect only those in the active period of sexual life. The first of these characteristics depends on the fact that tubercle, when it starts in the part most accessible to examination, the testis, owing to the loose nature of the structures here, is as a rule painless, and attention is not drawn to it till caseation is far advanced. The second is not easily explicable, but is shared by another disease of this tract—namely, gonorrhœa, to which the disease in question bears a certain relation. The third is due chiefly to our lack of knowledge of the serum therapy of tuberculosis, and to our inability to apply satisfactorily radical surgical methods to the deeply seated portions of this tract, as well as to the wide distribution of the disease in these portions. The last characteristic mentioned, although requiring no further explanation than that it is the nature of tuberculosis, wherever occuring, to affect young adult life, brings us to the consideration of the ætiology of genital tuberculosis.

In what manner does the tubercle bacillus gain access to these parts, and why does it more especially affect certain sections of them? It is obvious that it must arrive either by an indirect infection, *via* the blood stream, of some weak spot in the tract, the bacillus having been originally inspired or ingested; or that it must arrive per urethram by direct contagion.

If we look upon the first of these methods as the probable one, we must explain why the genital tract should be picked out as a particularly suitable nidus. Had any venereal disease occurred previously in a tuberculous patient to render these organs less resistant, the explanation would perhaps be a satisfactory one, and no doubt many cases do originate in this manner. But, on the other hand, many occur, as far as one can see, in patients who are not the victims of tubercle elsewhere and who have never had venereal disease; under these circumstances it would be unreasonable to exclude the possibility of the second method, that of direct contagion. Salleron points out that of fifty-one cases he examined, only one had evidence of tuberculosis elsewhere. The evidence in favour of local contagion is, however, not strong; v. Bramann denies that it ever occurs, but, on the other hand, such observers as Jacobson and Verneuil are of opinion that it does, and the former draws attention to cases reported by Zweigbaum and Kotschau, in which well-marked tuberculous lesions were present in the female genitals, in the former's case actually in the cervical canal; while Verneuil states a case in which a patient apparently devoid of tubercle or of tuberculous family history, developed the disease as the result of a gonorrhœal epididymitis. At the same time, in view of the non-motile character of the bacillus of tubercle and the fact that the urethra is an almost unknown seat of the disease, it is less likely that the contagion should be transmitted to the male from the female than that the reverse should occur, and it is known that the bacillus may be ejected in the seminal fluid of phthisical patients in whom the genital tract is apparently free from the disease.

In whichever way, then, the disease is implanted, there is as a rule some local determining cause: at times a blow, followed by a hydrocele; more often, perhaps, a precedent gonorrhœal epididymitis. At times none is to be found; thus I recently saw a young man in whom there was neither tuberculous family history nor, as far as could be discovered, evidence of tubercle elsewhere, but who had well-marked tuberculosis of the epididymis and right vesicula. This patient had not only never had gonorrhœa; he had never had sexual intercourse.

The question, then, must remain open; but it would be interesting to know, as the result of bacteriological research, in how many cases of uterine discharge the tubercle bacillus is present; and, in the case of such discharge in married women, how often tuberculosis is to be found in the genital tract of the husband. I cannot help thinking that were such an investigation to be carried out, the two conditions would be found to go hand in hand much oftener than is supposed.

Why does the tubercle bacillus select certain special sites in the genital tract? As is well known, the commonest situation for the deposition of the bacillus is the epididymis—usually the head, but at times the globus minor. Here the first evidence is to be found in the presence of a hard pea-like body, which consists of fibrous tissue, and which, on section, is seen to contain points of caseation. Presuming that the bacillus was carried to the epididymis by the blood stream, its arrest should be explicable either on anatomical or pathological grounds; thus it must be due to the smaller calibre of the epididymal vessels, or to the fact that these vessels have been constricted by some preceding fibroplastic lesion. Now, the statement that the disease commences in the epididymis before the testis is involved has been too readily accepted. It is certainly *noticed* earlier here, the lesions, when they exist in the testis, being less easily detected. In this connection Reclus has pointed out that in thirty-four cases examined post-mortem, both organs were involved in 75 per cent. of the number.

My own experience, derived both from testes examined after excision by operation and in the post-mortem room, certainly suggests that in an almost equal number of cases the disease will be found in both testis and epididymis, although there may be no outward sign of it in the former.

Again, supposing that the disease be derived by direct inoculation, how does it come that the bacillus avoids the urethra entirely, passes the prostate and ejaculatory ducts and the seminal vesicles, and finally lodges in the lower end of the vas deferens? Since its activity is favoured by the ravages of the gonococcus, a fact which has been pointed out by Kocher and Simmonds, we should expect to find the urethra, and especially the bulbous portion of it, to be frequently the seat of tuberculosis, yet this is not so. We must, however, remember that tubercle can rarely resist an actively acute inflammation like gonorrhœa, and that what happens is that the bacillus finds a nidus, not in the seat of acute inflammation, but in the damaged portion after that inflammation has subsided. But this portion is not the urethra: Strumpell has pointed out that, just as in inhalation tuberculosis of the lung, where the disease avoids the trachea and bronchi and settles in the pulmonary alveoli, so in the genital tract it avoids the urethra and settles in the epididymis. The bacillus would seem to seek out those parts where it is undisturbed, in the one case by the passage of strong currents of air, and in the other by that of constantly recurring streams of urine.

Certain writers, among whom Konig may be mentioned, maintain that the epididymis is not the first seat of origin, and that this doubtful honour must be accorded to the prostate; this is not, however, the experience of most observers; and Baumgarten, who made experiments with animals, found that injection of tuberculous material into the urethra, although it produced tubercle of the base of the bladder and prostate, never affected the testis or kidney. On the other hand, injection of the bacillus into the testis resulted in the invariable production of tubercle in the corresponding vesicle and prostate even after ligation of the vas deferens. These facts led him to suppose that infection goes always with the stream of secretion and lymph, and never against it. Personally I can recall no case in which the disease appeared to originate in the prostate or vesiculæ. In the great majority of cases, if not in all, tubercle commences in the epididymis or testis, and to these points it may remain localised for many weeks or even months. But it must be remembered that tubercle is a disease that never remains stationary; it either gets well or else it gets worse, and while it appears to be stationary, it is progressing towards the stage of caseation, which is the parting of the way either towards suppuration or resolution by fibrosis. In course of time other nodules will appear in the epididymis, and these coalescing with the main deposit, the whole organ becomes converted into a hard nodular mass.

Before proceeding further let us consider the diagnosis of tuberculosis of the epididymis in its early and late stages. Such a nodule as I have mentioned may be closely simulated by a tense cyst either of the epididymis itself, or arising from the hydatid of Morgagni or the vas aberrans of Haller. Here is a hard nodule which may or may not be tuberculous: in the former case, it is in the stage in which its further progress must be rendered impossible; it has been noticed accidentally by the patient, who probably does not know exactly how long it has been present; on what does the diagnosis depend? At this stage there is very little to help one: these cysts are, however, usually devoid of pain; they feel rather on the surface than in the substance of the epididymis. Occasionally they are translucent, but in the ordinary way this sign is difficult to obtain; it is most easily sought for by means of such a lamp as is used for the illumination of the frontal sinus. At this stage too it is not common to find a hydrocele present, and, therefore, the two most valuable signs at your disposal are the lack of translucency and the presence of definite tenderness.

The next stage is that in which the whole epididymis is involved, and on palpation is felt to be thickened and fibrous. Here the diagnosis may be difficult because the condition so frequently follows a gonorrhœal epididymitis, which, as it subsides, leaves the organ indurated and tender for a considerable time. Both these conditions are accompanied by hydrocele to some extent, and it is possible for them to be present at the same time. Now it is at this stage of the disease that we expect to find the evidences of its further extension, and it rarely progresses thus far without such. Commonly the structure next involved is the vas deferens, and this will be found to be affected in two different ways. Either solitary nodules will be found, usually opposite the head of the epididymis or just above the vesiculæ seminales, the remainder of the vas being unaffected; or, in more acute cases, the disease will progress by a steadily ascending infiltration, the whole vas being gradually converted into a thick rigid tube. It is in such cases that the vesiculæ are almost sure to be found affected, and then the diagnosis is obvious. But supposing nothing of the sort is present, three methods of arriving at a conclusion are open to us. Either we can wait for further developments, or we can explore by open incision, or we can utilise Calmette's reaction.

The first of these methods is mentioned only to be severely condemned. Tubercle is a disease which, in its early stage, is in this situation most accessible, but in its later stages quite the reverse; it is a disease which, if it cannot be arrested, must eventually lead to the death of the patient; in any case, unless taken without delay it is almost certain to attack the other testis and epididymis.

Exploration is unsatisfactory, firstly, because the naked eye appearance of the testis and epididymis in the early stages of tubercle is much the same as that in chronic gonorrhœal epididymitis, and, secondly, because of the danger of stimulating the disease by interference, and possibly causing adhesion of the epididymis to the skin wound where the exploration has failed to clear up the diagnosis, especially if the fibrous capsule of the organ has been damaged.

It is a little difficult to form an accurate opinion of the value of Calmette's reaction in view of the varying accounts which have been given of its effects. A review of these accounts, however, leads one to suppose that it is only reliable in quite early cases of tuberculosis; that it is not absolutely reliable even in these cases; and that there is some reason to fear permanent damage to the eye, or at least serious temporary derangement, by the excess or persistence of the inflammatory reaction set up. Much work must be done before a definite conclusion can be arrived at as to its real value.

Within a certain period, at times quite early in the case, the infection spreads to the body of the testis, and the arrangement of the deposits, which are chiefly seen towards the periphery of the organ, would suggest that this infection is brought about through the medium of the blood stream, and is not always the result of local extension from the epididymis. The latter, however, also occurs, as is shown by the presence of the bacillus in the tubules of the testis, and by the fact that the largest areas of caseation are found in the corpus Highmori, the hilum of the testis. The probability is that in those cases in which the infection of the testis is early the deposit is determined by the blood stream at the same time as in the epididymis, though not noticed till later; and that in those in which minute deposits in the testis are coincident with extensive disease of the epididymis, the infection is the result of direct extension from the latter *via* the vasa efferentia.

It is interesting to note that the tunica vaginalis is at times involved when there is no evidence of disease in the testicular body. It may present three distinct conditions. Thus it may simply contain fluid, not usually in large quantity; it may be much thickened by inflammation and adherent to the body of the testis; or it may present the less common condition described by Gerster, in which it closely resembles a tuberculous synovial membrane.

When the disease commences primarily in the testicular body the diagnosis may at times be very difficult, since, at all events at first, it may closely simulate certain other diseases of the organ. Thus it may be mistaken for syphilitic orchitis, either interstitial or gummatous, or for commencing malignant disease; and the resemblance may at first be very close. It is met with in two different varieties, the one a chronic gradual enlargement of the organ, usually slightly tender and having testicular sensation fairly maintained; two points, it will be noticed, which serve to distinguish it from secondary syphilitic orchitis and growth; and the other, a rapid general enlargement of the testis and epididymis, or of the testis only, accompanied by acute pain, swelling, and inflammation of the scrotum, and at times the discharge of sero-purulent matter from the urethra which is not gonorrhœal. This latter variety is rare, and is easily confounded with an epididymo-orchitis of gonorrhœal origin. It, however, generally ends in a short time in suppuration and the discharge of caseous material, and complete destruction of the testis, which, of course, is an occurrence of extreme rarity in gonorrhœal orchitis.

In the commoner of these two varieties difficulty is only likely to arise in the complete absence of other signs of the diseases mentioned; in such cases the diagnosis may be materially simplified, provided that any degree of hydrocele is present, for it has been shown by Tuffier that hydrocele fluid from such cases, when injected into the peritoneal cavity of animals, results in fatal tuberculosis, even when no bacilli have been demonstrated in it.

With regard to the vesiculæ seminales and prostate, the general consensus of opinion now seems to be in favour of the belief that disease here is never primary, but always secondary, either to tuberculosis ascending from the testis or descending from the kidney; at the same time one must not forget that Baumgarten's experiments point to the possibility of such an occurrence, and if it actually occurs in the human being, go far in support of the theory of local inoculation during coitus.

The infection of these organs is usually a later phase of the disease, and occurs, as a rule, about the same time. Unless the prostate is extensively diseased, the subjective symptoms are not very marked; but if caseation and cavitation have advanced to any extent, the bladder will usually be found to be in a state of ulceration about the trigone, and it is to this rather than to any change in the prostate that the symptoms—namely, burning pain, tenesmus, and strangury—are due. The enlargement of these organs in tuberculosis is not usually very great, but they become markedly nodular and hard, especially in the case of the prostate, which is more easily felt per rectum than are the more unsupported vesiculæ. When doubt exists as to involvement of the prostate or vesicles, it may be possible in certain cases to express from them into the urethra tuberculous detritus which can then be examined.

A very important matter with regard to the progress of the disease is the question of how far the second testis is likely to become affected; when is this infection likely to occur, and can we reasonably expect to prevent it? There is no doubt that a very large proportion of cases eventually run on to bilateral infection, and Hass's statistics, which place this occurrence after unilateral excision at 25 per cent., are, I think, unduly optimistic. Whether the infection is carried by the blood stream or results from local contagion per urethram, it is difficult to see why one testis alone should be affected; it is, I think, more reasonable to suppose that the second organ should be infected at or about the same time and in the same manner as the first, than that the first should be the only one affected through paths of infection which are equally shared by both organs, and should involve its fellow by the much more round about method of upward extension along the vas deferens as far as the urethral orifices of the ejaculatory ducts, and then downwards through the vas of the opposite side. Infection of the second organ is unfortunately very probable, and this is especially so where it has been the seat of old gonorrhœal trouble. The time at which the second testis is discovered to be affected, however, varies greatly; sometimes it will be seen to be diseased quite early in the case, but more usually about the time that nodules are first felt in the prostate and vesiculæ. The opinions of other observers with regard to bilateral infection are worth noting. For instance. v. Bramann states that the statistics of the Berne clinic show that the disease becomes bilateral in 75 per cent. of cases; Bruns that in 78 cases of unilateral castration the other testis eventually became involved in 23 per cent.; while Godlee, in his Bradshaw lecture of last year, frankly owns that he has "given up removing every tuberculous testicle, however quiescent, if no sign of tubercle could be found elsewhere, because it has so often led to disappointment, the patient returning before long with the opposite testicle affected."

It will be seen from what I have said that the whole question as to infection, direction of propagation, and subsequent involvement of the other side, is still open ground for discussion, and opinions on each of these points can be quoted which will be found to differ materially from each other.

The treatment of tuberculosis of the genital tract is, or should be, from the first entirely surgical, for although we know that in other parts of the body, and, indeed, even in this very part, careful treatment on palliative and hygienic lines may at times result in the resolution of the early lesions of tuberculosis, yet we cannot guarantee such a result in any particular case. If in the situation we are considering such treatment should fail, the time of grace for radical treatment slips through our fingers, and it is the patient, and not we, who pays forfeit, possibly with his life. To quote Jacobson, "tubercular disease should be looked upon as malignant, and treated as such, not in a way which trifles with it and courts recurrence."

In the earliest stage of the disease, then, when yet as far as one can tell the trouble is confined to the epididymis, radical measures are even more surely indicated than in those cases in which the disease is more advanced. The diseased organ must be removed, or at least the diseased portion of it must be destroyed. Now half measures are here worse than useless, and therefore I would say, avoid such operations as scraping and cauterising; they are only permissible where the trouble is hopelessly distributed, and where any more drastic proposal is met by an obstinate refusal on the part of the patient or his relatives. The question then is, will epididymectomy suffice, or must the whole testis be excised?

At first sight it would appear to be reasonable to submit the patient to the lesser operation, and so retain the internal secretion of the testis for his benefit; but when it is remembered that it is impossible to be certain of the integrity of the testis, that the vas deferens is often diseased at the level of the seminal vesicles before anything can be felt there, that even when the testicular body itself is intact the tunica vaginalis may contain tuberculous hydrocele fluid, and lastly that the power of internal secretion is exercised to the full as easily by one organ as by two, it must be obvious that the operation of epididymectomy is very closely allied to those procedures generally known as "tinkering." If the operation has any proper status in these cases, it is, I think, in that in which one testis has already been sacrificed, and in which the patient objects to parting with the other; or possibly where both epididymes are involved, and the operation is done with a view to preventing the occurrence of scrotal abscesses. If we follow the advice given by Jacobson and treat the disease as if it were malignant, nothing short of complete excision of the whole organ, with as much of the vas deferens as can be secured, can satisfy the requirements of sound surgery. Czerny and Bruns, to quote two of the highest authorities, go so far as to say that in view of the frequent involvement of the testis of the other side, double castration is the operation of election. To this I think most British surgeons would object, and, I think, rightly so. For if, after unilateral excision and extirpation of the vas, the disease appears in the other testis, it would seem to suggest either that the second organ was affected already at the time of operation; that the disease was conveyed from a point beyond the limits of the operation—namely, the vesiculæ or prostate; or that the infection is an independent secondary deposit by the blood stream. In the first and second of these instances the disease was obviously beyond cure, while the employment of such a drastic remedy as double castration to prevent the third seems to me rather like chopping off a man's head to prevent his contracting cancer of the tongue.

So far, then, castration, or rather unilateral excision of the testis and extirpation of the vas deferens, would seem to be the most rational treatment, but to be successful it is essential that it should be undertaken in the earliest stages of the disease. If castration proper is to be retained as a recognised operation in these cases, it should be confined to the later stages, and performed, not with a view to cure, but as a means of ridding the sufferer of a mass of tissue, which, if left to itself, will result in the formation of deep and painful sinuses in the scrotum and groins, constantly bleeding and discharging, and reducing him to a condition of exhaustion and misery.

Whether unilateral excision or castration is in contemplation, it is important that it should be undertaken before the disease has attacked the skin of the scrotum, since, apart from the danger of tuberculous infection of the skin, it must be remembered that the removal of a testis which is adherent to its surroundings involves much persistent oozing, which encourages sepsis; and where there is already a sinus present, the resulting sepsis, running as it does in planes of tissue which are loose and lymphatic, is very difficult to limit, and in wasted patients is prone to result in serious consequences. With regard to extirpation of the vas deferens from the inguinal region there are one or two points of importance. The lower end of this tube is very deeply placed, and requires a wide incision to reach it; this incision should lie along the inner two-thirds of Poupart's ligament, and should divide the abdominal aponeurosis and separate away the ligamentous origin of the internal oblique muscle. Being then widely retracted, the peritoneum must be raised from the iliac fossa, the deep epigastric artery being tied in two places and divided if necessary. In this way the extreme lower end of the vas may be reached; it should on no account be torn out, as has been suggested, since in doing this it may give way at a point of infection and so distribute the disease, or it may cause bleeding which will be almost impossible to control. It should be double ligatured and divided, the proximal end being touched with pure carbolic acid, after which the wound must be treated as in Bassini's operation for inguinal hernia.

There is still some difference of opinion as to the advisability of attempting the removal of the vesicula seminalis of the same side, where unilateral excision is performed. The majority of surgeons are, I think, against it, and although I was at one time in favour of it, I confess frankly that I have found it disappointing and I have abandoned it. The objections to it are five in number. In the first place it is a prolonged and certainly not an easy operation, owing to the depth at which the vesicula lies and to its sessile and unsupported character; secondly, the disease, when present at this level, has almost certainly passed to the opposite side, although not palpable; thirdly, so deep a wound in such close proximity to the anus is liable, if infected, to suppurate, and so to form a sinus of a very intractable type; fourthly, when very adherent to the bladder through chronic inflammation of its capsule, there is a not inconsiderable probability of opening that viscus; and lastly, if by any chance the vesicula is ruptured in removal, the cellular planes about the base of the bladder and around the rectum form the most ideal nidus for the development of the tubercle bacillus. There is no doubt that it is a great temptation to remove the vesicula when the only evidence of disease is a nodule in the epididymis and some thickening of the vesicula. I am, however, more convinced than ever that such treatment will end in disappointment, and will not prevent the second testis from becoming involved.

Returning to the question of castration, it is well to bear in mind the possible after effects of the operation, and that on no account should it be done without the full knowledge on the part of the patient of these effects, and without his consent or that of his parents. At the same time, it should be made clear that such effects are by no means common. Of greatest importance is the alteration in the male attributes of the patient when the operation is performed at or before the age of puberty: the arrested development of the growth of hair on the face, and the retention of the soprano voice must be expected at this period; it is, however, unusual to find such phenomena in patients who have undergone the operation after the full development of the male characteristics. In later life, especially in approaching old age, there is some danger of the operation being followed by mental changes, chiefly in the direction of imbecility. This sequela was frequently noticed at the time when it was the custom to perform the operation for enlargement of the prostate; and of three cases in patients of the age of seventy to seventy-five of which I have knowledge, two of them became completely imbecile within three months of the performance of the operation. As, however, tuberculosis of the genital tract affects more especially patients between the ages of eighteen and thirty-five, such complications are seldom seen.

Lastly, the question must be considered (and is, indeed, at times put to one professionally) as to how far it is justifiable for a patient who has undergone unilateral excision of the testis to marry? Those of us who have keenly at heart the burning question of the causes of deterioration of the national physique will be at no loss to answer this. In view of the fact that tuberculosis is a disease largely depending on hereditary susceptibility, and is therefore liable to break out afresh at various periods of life; that the testis is a part which is very prone to be attacked; that the disease here may be latent for long periods; that the virus is easily communicable at the moment of seminal emission; and that the act of procreation under such circumstances inflicts an incalculable wrong, not only on the innocent offspring of such a union, but also on the national heritage of which it forms a part, it is easy to see that such marriages are not only unjustifiable, they are worse, for they are cruel and immoral.

